# Accelerated Mammary Tumor Onset in a HER2/*Neu* Mouse Model Exposed to DDT Metabolites Locally Delivered to the Mammary Gland

**DOI:** 10.1289/ehp.1104327

**Published:** 2012-04-18

**Authors:** Nakpangi A Johnson, Arline Ho, J. Mark Cline, Claude L Hughes, Warren G Foster, Vicki L Davis

**Affiliations:** 1 Graduate School of Pharmaceutical Sciences, Duquesne University, Pittsburgh, Pennsylvania, USA; 2 Cedars–Sinai Medical Center, Los Angeles, California, USA; 3 Department of Pathology, Wake Forest University Medical Center, Winston–Salem, North Carolina, USA; 4 General Medicine Therapeutic Delivery Unit, Quintiles, Research Triangle Park, North Carolina, USA; 5 Department of Obstetrics and Gynecology, McMaster University, Hamilton, Ontario, Canada

**Keywords:** antiandrogen, breast cancer, DDT, endocrine disruptor, HER2/*Neu*, *o,p*´-DDE, *p,p*´-DDE

## Abstract

Background: The association of DDT (dichlorodiphenyltrichloroethane) with breast cancer is controversial, but animal studies directly linking DDT to risk are lacking. Concerns with DDT reside in its environmental persistence, bioaccumulation in breast adipose tissue, and endocrine-disrupting actions. Whereas most attention has been focused on estrogenic congeners, we tested the cancer-inducing potential of the antiandrogen, *p,p*´-DDE [1,1-dichloro-2,2-bis(*p*-chlorophenyl) ethylene], the most prevalent and persistent DDT metabolite.

Objectives: We aimed to determine whether developmental exposure to *p,p*´-DDE stored in adipose tissue surrounding the cancer-prone mammary epithelium of MMTV-*Neu* mice influences tumor development.

Methods: For localized delivery, Elvax 40P pellets containing *p,p*´-DDE were implanted into the mammary fat pads of prepubertal female mice. We compared mammary tumor development with *p,p*´-DDE with development in response to its estrogenic isomer, *o,p*´-DDE [1,1-dichloro-2-(*o*-chlorophenyl)-2-(*p*-chlorophenyl) ethylene], and a mixture of both isomers.

Results: *p,p*´-DDE implants significantly accelerated mammary tumor onset compared with vehicle Elvax implants. *o,p*´-DDE had similar results, but only at ≤ 10 months of age. Lipid-adjusted levels of *p,p*´-DDE in mammary adipose tissue and serum in young mice were within the ranges of human exposure, whereas concentrations in aged mice were low to undetectable. Exposure to a 2:1 ratio of *p,p*´-DDE:*o,p*´-DDE did not result in the younger latency observed with the individual isomers.

Conclusions: *p,p*´-DDE exposure at concentrations relevant to human exposure accelerates mammary carcinogenesis in mice, possibly through hormonal and/or other actions. These data suggest that DDE exposure would promote, but not cause, mammary tumorigenesis. Developmental exposure in immature mammary tissue continues to affect tumor onset even after *p,p*´-DDE levels have declined. Future studies are needed to determine whether early exposure to *p,p*´-DDE correspondingly predisposes women to early-onset breast cancer.

Even with its use banned in the United States for decades, the pesticide DDT (dichlorodiphenyltrichloroethane) has sparked considerable concern pertaining to breast cancer because of its abundance and persistence in the environment, its ability to bioaccumulate in breast adipose tissue, and its endocrine-disrupting activities (Snedeker 2001). Most of the concern with DDT exposure has centered on estrogenic congeners, including *o,p*´-DDT and *o,p*´-DDE [1,1-dichloro-2-(*o*-chlorophenyl)-2-(*p*-chlorophenyl) ethylene], and little focus has been directed toward the antiandrogen, *p,p*´-DDE [1,1-dichloro-2,2-bis(*p*-chlorophenyl) ethylene]. *p,p*´-DDE binds to the androgen receptor and inhibits androgen responses *in vitro* and *in vivo* with equal efficacy as the potent antiandrogen hydroxyflutamide (OH-flut) (Kelce et al. 1995). Because androgens can diminish the stimulatory effects of estrogens on mammary tissue (Somboonporn and Davis 2004), an antiandrogen could counteract this protection and stimulate proliferation of normal mammary epithelium as has been reported with flutamide treatment in rhesus monkeys (Dimitrakakis et al. 2003). Therefore, exposure to *p,p*´-DDE may adversely affect mammary carcinogenesis.

Many studies report no correlation between higher *p,p*´-DDE levels in aged women and breast cancer risk (Lopez-Cervantes et al. 2004; Shakeel et al. 2010; Snedeker 2001); however, the unknown timing of initial exposure and the levels of exposure at younger ages may obscure evidence of DDT’s impact in older women. In contrast, young women with higher *p,p*´-DDT levels had a significant 5-fold increase in breast cancer risk when they were ≤ 14 years of age at their first possible DDT exposure (Cohn et al. 2007). These findings suggest knowledge of exposures at younger ages may be essential for assessing breast cancer risk.

In the present study, we tested the premise that *p,p*´-DDE influences mammary tumor development using the unactivated MMTV-*Neu* mouse model (Guy et al. 1992). The advantage of testing the effects of *p,p*´-DDE in an animal model is the ability to control for the unknowns and variables that occur in epidemiological studies such as the initial age of exposure, early levels of exposure, diet, genetics, and environment, thereby allowing a direct correlation between mammary tumor outcomes and DDE exposure to be established. One potential mechanism for *p,p*´-DDE affecting mammary carcinogenesis is through its antiandrogenic actions. *o,p*´-DDE was examined for comparison because of its estrogen-like actions and similar chemical structure. Although the resulting mammary tumors are estrogen-receptor (ER)–negative, tumor development in this model is estrogen dependent (Menard et al. 2000), thereby allowing investigation of potential estrogenic influences by the DDE isomers. The speculation surrounding the influence of DDT on breast cancer resides in the high levels of its endocrine-disrupting congeners, which accumulate in the breast with the potential to modify the local hormone environment (Snedeker 2001). To determine whether the DDE stored in mammary adipose tissue directly affects the neighboring cancer-prone epithelium, we examined the effects of localized delivery of DDE using Elvax 40P pellets implanted into the mouse mammary glands. Elvax 40P is an ethylene vinyl-acetate copolymer that causes no inflammatory response and is capable of sustained, local release of macromolecules *in vivo* (Silberstein and Daniel 1982). In the mammary gland, Elvax implants containing 17β-estradiol (E_2_) are reported to cause local estrogen responses without systemic effects (Haslam 1988). This form of delivery allows for slow, local accumulation of lipophilic DDE to mimic the gradual accumulating exposure in breast fat in women. As DDT exposure can occur before birth (Foster et al. 2000) and early exposures may have more impact than exposures in the adult breast (Cohn 2011), we initiated DDE treatment in prepubertal mice to determine whether developmental exposure to DDE isomers with antiandrogenic and estrogenic activity influences tumor formation.

## Materials and Methods

*Animals.* Animals were treated humanely and with regard for alleviation of suffering according to approved protocols by the Cedars–Sinai Institutional Animal Care and Use Committee and National Institutes of Health guidelines. Hemizygous female MMTV-*Neu* mice harboring the *Neu* protooncogene (FVB/N-Tg(MMTVneu)202Mul/J) (Guy et al. 1992) were bred from dizygous MMTV-*Neu* males and FVB/N (wild-type) females (Jackson Laboratory, Bar Harbor, ME, USA). The breeders and progeny were maintained on an isoflavone-free diet (a modification of AIN-93G using corn oil with 20% protein, 16% fat, and 64% carbohydrates; Harlan-Teklad, Madison, WI, USA) to prevent phytoestrogen exposure during all developmental stages of the study mice.

*Localized treatments in mammary glands.* Treatment pellets were prepared using Elvax 40P (provided by Dupont, Wilmington, DE, USA) as previously described (Silberstein and Daniel 1982). The treatment stocks for *o,p*´-DDE and *p,p*´-DDE (Crescent Chemical Co., Islandia, NY, USA), E_2_ and [17α]-methyl testosterone (Sigma-Aldrich Co., St. Louis, MO, USA), and OH-flut (provided by R. Neri at Schering-Plough, Kenilworth, NJ, USA) were dissolved in 95% ethanol for addition to Elvax 40P during pellet preparation. Elvax pellets containing the test chemicals or vehicle (95% ethanol) were implanted through a surgical incision on each flank into each axillary (gland 3) and inguinal (gland 4) mammary fat pad (4 pellets/mouse) at weaning (mean age 21.7 days) under isoflurane anesthesia. Because MMTV-*Neu* tumor formation is stochastic (Guy et al. 1992), pellets were implanted into each fat pad.

*Study mice.* Hemizygous MMTV-*Neu* female mice were weaned and randomized into seven treatment groups (control with vehicle implants, *n* = 74; 5-μg/pellet *p,p*´-DDE, *n* = 80; 5-μg/pellet *o,p*´-DDE, *n* = 73; 2:1 ratio of *p,p*´-DDE:*o,p*´-DDE, total 5 μg/pellet, *n* = 73; 2.5-ng/pellet E_2_, *n* = 78; 50-ng/pellet methyl testosterone, *n* = 74; and 5-μg/pellet hydroxyflutamide, *n* = 78) before pellet implantation. Tumor onset was determined by weekly palpations from 2 months of age until a maximum age of 14 months. Animals that died early unrelated to tumor formation or unconfirmed to be tumor-free after death (approximately 22%) were excluded from the incidence analysis but not from survival curves unless death occurred before initiating palpations. No significant differences in excluded mice were detected between the groups (*p* > 0.05, chi-square test). Mice with maximum tumor burden, tumors affecting mobility or ulceration, or illness were euthanized before reaching maximum age (approximately 21%). Histopathological assessments of metastatic cancer incidence in hematoxylin and eosin–stained sections of mouse lungs by our board-certified veterinary pathologist (J.M.C.) was performed as previously described (Davis et al. 2008). Real-time polymerase chain reaction for *Neu* expression was performed as previously described with primers specific to the mouse and rat (transgene) *Neu* genes (Davis et al. 2008).

*DDE concentrations in serum and mammary fat pads.* Residue concentrations of *o,p*´ and *p,p*´ isomers of DDE, DDT, and DDD (dichlorodiphenyldichloroethane) were measured in blinded serum and mammary adipose tissue samples by the Institut National de Santé Publique du Québec (National Public Health Institute of Quebec; Quebec, Canada) following established procedures (Demers et al. 2000). Briefly, the samples were spiked with the *p,p*´-DDE internal standard, denatured in formic acid, extracted with solid phase separation, and cleaned on a Florisil column before gas chromatography–mass spectroscopy (GC-MS) as described in Supplemental Material, p. 3 (http://dx.doi.org/10.1289/ehp.1104327). Randomly selected individual samples from *p,p*´-DDE, *o,p*´-DDE, 2:1 ratio, and control groups were pooled from young mice treated for 2 months (one pool, *n* = 12) and aged animals (three pools, *n* = 5/pool) to result in at least 500 mg or 1 g of mammary adipose tissue for the young and old mice, respectively, and 2 mL of serum for both ages; the same animals were examined for both tissues. Total serum lipids or percent lipids in mammary fat pads were measured by enzymatic methods to calculate lipid-adjusted concentrations (nanograms per gram lipid) (Bernert et al. 2007). Our reported limits of detection (LODs) vary between samples because of adjustments for lipid concentrations.

*Statistics.* We used the chi-square test for analyzing categorical variables, one-way analysis of variation (ANOVA) with Tukey’s post-test for comparing means between multiple groups, and the Gehan–Breslow–Wilcoxon test for survival curves (because it puts more weight on early events and requires one group to have a consistently higher risk than the other, as would occur with a shorter latency). Analyses were performed using GraphPad Prism 5.0 software (GraphPad Software Inc., San Diego, CA, USA) with *p* < 0.05 considered significant.

## Results

**Figure 1:**
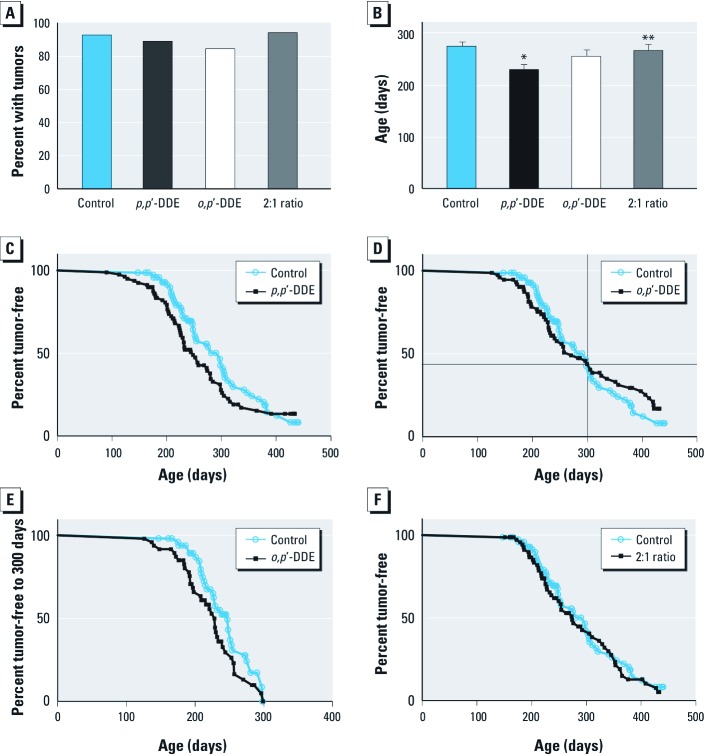
Localized delivery of *p,p’*-DDE and *o,p’*-DDE accelerates mammary tumor development in MMTV-*Neu* female mice. (*A*) No significant difference in maximal mammary tumor incidence between the groups was detected (*p *> 0.05, chi-square test) for tumors detectable by palpation and/or at necropsy in all mammary glands (control, *n *= 57; *p,p’*-DDE, *n *= 65; *o,p’*-DDE, *n *= 59; and 2:1 ratio, *n *= 54). (*B*) Tumor onset determined by weekly palpations was significantly different for the four groups (*p *= 0.0087, one-way ANOVA) and between the *p,p’*-DDE and control and 2:1 ratio groups by Tukey’s test (mean ± SE is shown; control, *n *= 53; *p,p’*-DDE, *n *= 58; *o,p’*-DDE, *n *= 50; and 2:1 ratio, *n *= 51). (*C*) Survival curves for the percentage of animals that are tumor-free at the designated ages for the control and *p,p’*-DDE (5 μg/pellet; 4 pellets/mouse) groups were significantly different (*p *< 0.02, Gehan–Breslow–Wilcoxon test) with the *p,p’*-DDE curve shifted to younger ages. (*D*) The *o,p’*-DDE (5 μg/pellet; 4 pellets/mouse) and control survival curves were not significantly different when all ages (≤ 14 months of age) were assessed (Gehan–Breslow–Wilcoxon test). (*E*) The *o,p’*-DDE survival curve was significant compared to the control group when the curves only up to 300 days of age were analyzed (*p *< 0.05, Gehan–Breslow–Wilcoxon test), the age when the *o,p’*-DDE curve crossed the control curve in panel D. (*F*) The 2:1 ratio curve (5 μg/pellet; 3.3 μg *p,p’*-DDE + 1.7 μg *o,p’*-DDE; 4 pellets/mouse) resembled the control group (*p *> 0.05, Gehan–Breslow–Wilcoxon test). *p < 0.05 compared with control. **p < 0.05 compared with *p*,*p*’-DDE.

*Mammary tumor development with localized DDE exposure.* The maximal incidence of mammary tumors after localized delivery of 5 μg *p,p*´-DDE via Elvax pellets to the four mammary fat pads (20 μg/mouse) was not modified compared with the control group implanted with vehicle pellets (Figure 1A). However, the age of tumor onset was significantly earlier with *p,p*´-DDE treatment (*p* = 0.0008, *t*-test), by approximately 1.5 months compared with control mice (Figure 1B). The earliest tumor was detected in the *p,p*´-DDE group almost 2 months before the first tumor in the control group (age 90 vs. 147 days). Survival curves depicting the percent of tumor-free mice with age verify that prepubertally initiated *p,p*´-DDE treatment consistently resulted in accelerated mammary tumor formation (Figure 1C).

Unlike *p,p*´-DDE, exposure to estrogenic *o,p*´-DDE (5 μg/pellet) did not result in an earlier latency (Figure 1B), and maximal incidence was not significantly different compared with control or *p,p*´-DDE groups (Figure 1A); however, the first tumor was detected early (126 days). Because the survival curve shows tumor onset was accelerated before 300 days of age (10 months) and delayed after this age (Figure 1D), the latency and survival curves for *o,p*´-DDE were not significantly different compared with control mice when both age ranges were examined (Figure 1A and 1D). However, when we evaluated the first 300 days separately, the curve for the *o,p*´-DDE group was significantly shifted left (Figure 1E), demonstrating an earlier tumor onset.

Because DDT contamination in women occurs as a mixture, another group of mice was treated with both *p,p*´-DDE and *o,p*´-DDE. A 2:1 ratio of *p,p*´-DDE:*o,p*´-DDE was tested at the total concentration of 5 μg/pellet. The mixture was designed to determine whether the two isomers had similar or additive (hormonal or non-hormonal) affects on mammary tumorigenesis while keeping a higher concentration of *p,p*´-DDE, as *p,p*´-DDE is the most prevalent DDT contaminant in humans. As expected, maximal incidence was not modified by the 2:1 ratio. Although both individual isomers accelerated tumorigenesis, the 2:1 ratio latency was not different from the control or *o,p*´-DDE groups and was significantly later than the *p,p*´-DDE–induced early onset (Figure 1B). No effect on mammary tumor development was observed as survival curves for the 2:1 ratio and control groups were similar (Figure 1F).

*DDE concentrations in mammary fat pads and serum.* In young mice treated 2 months with *p,p*´-DDE, mammary adipose tissue contained 1,800 ng/g lipid (i.e., 1,800 μg/kg or ppb lipid) *p,p*´-DDE (Table 1). At this age, the implants should still be releasing the treatments based on the expected 100-day delivery for Elvax pellets (Silberstein and Daniel 1982). Because of its ubiquitous environmental presence, it was not unexpected to detect *p,p*´-DDE in control mice. In the *o,p*´-DDE group, the concentration of *o,p*´-DDE was lower than *p,p*´-DDE despite delivery of the same dose in both groups. Serum concentrations were lower than in fat, as expected; however, the ratio of serum:adipose levels was considerably lower for *o,p*´-DDE (1:136) compared with *p,p*´-DDE (1:8). For the 2:1 ratio group, both isomers were detectable in serum and mammary fat, although not proportionally equivalent to the concentrations in the single isomer pellets (3.3 + 1.7 vs. 5 μg/pellet).

**Table 1:** Lipid-adjusted DDE concentrations in mammary adipose tissue and serum in young MMTV-Neu female mice after 2 months of treatment.

DDE isomer/treatment group			Serum (ng/g lipid)a	Mammary fat pad (ng/g lipid)a
p,p’-DDE					
	Control		(< 14)		12
	p,p’-DDE		230		1,800
	o,p’-DDE		(< 15)		11
	2:1 ratio		150		1,600
o,p’-DDE					
	Control		(< 1.6)		(< 2.4)
	p,p’-DDE		(< 1.6)		(< 2.2)
	o,p’-DDE		8.8		1,200
	2:1 ratio		3.4		590
aOne pool/group (n = 12/pool); for samples < LOD, the lipid-adjusted LODs are shown in parentheses.					

After pellet depletion in aged mice and no additional exposure, mammary fat pad and serum DDE levels were substantially lower (Table 2) compared with young females (Table 1). In serum, the concentrations were near or below LODs for both isomers. In contrast, mammary adipose concentrations were detectable only for *p,p*´-DDE (Table 2). *p,p*´-DDE concentrations in the 2:1 ratio group were significantly higher than the isomer alone, despite its lower dose in the pellets. These higher concentrations suggest that the mixture may enhance retention of *p,p*´-DDE in adipose tissue. The concentrations of the other DDT congeners, *p,p*´-DDT, *p,p*´-DDD, *o,p*´-DDT, and *o,p*´-DDD, were not detectable in either tissue at either age (data not shown). The mouse ages were ≥ 9 months (one 2:1 ratio pool had a 6.5-month-old mouse, but it had the lowest concentration of the three pools for both tissues); thus, *o,p*´-DDE adipose concentrations were < LOD in the aged mice around the age at which the earlier onset of tumors ended (Figure 1D).

**Table 2:** Lipid-adjusted DDE concentrations in aged MMTV-Neu mice from mammary adipose tissue and serum.

DDE isomer/treatment group			Average age (months)^a^	Serum (ng/g lipid)^a^	Mammary fat pad (ng/g lipid)^a^
p,p’-DDE							
	Control		13.0 ± 0.5		(< 18)b		5.1 ± 0.6
	p,p’-DDE		11.6 ± 0.5		(< 18)b		12.2 ± 3.6
	o,p’-DDE		11.8 ± 0.5		(< 10)b		3.9 ± 0.3**
	2:1 ratio		11.4 ± 0.6		12c		21.7 ± 2.7*
o,p’-DDE							
	Control		13.0 ± 0.5		1.2c		(< 2.5)b
	p,p’-DDE		11.6 ± 0.5		(< 2.0)b		(< 2.2)b
	o,p’-DDE		11.8 ± 0.5		1.2c		(< 2.3)b
	2:1 ratio		11.4 ± 0.6		1.4c		(< 2.2)
Values are mean ± SE except as indicated and represent three pools/group (n = 5/pool). aThe LODs after adjusting for lipids are shown in parentheses for pools < LOD for all three pools tested. bFor groups with varying LODs, the pool with the highest limit is shown. cOnly one of the three pools was > LOD. *One-way ANOVA, p = 0.002 for the four groups analyzed for p,p’-DDE levels, n = 3; Control vs. 2:1 ratio significant by Tukey’s post-test (p < 0.01). **o,p’-DDE vs. 2:1 ratio significant by Tukey’s post-test (p < 0.01).							

**Figure 2:**
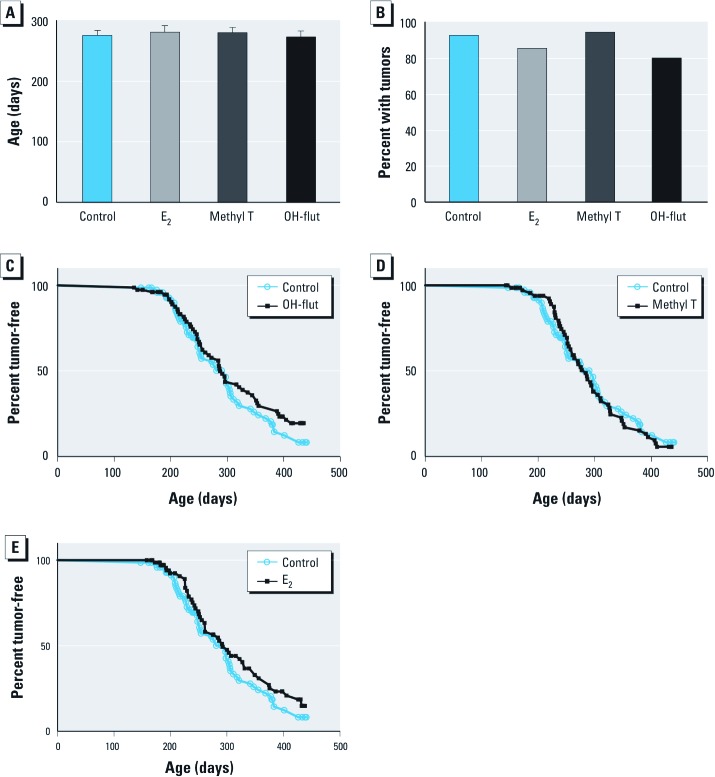
The E_2_, methyl T, and OH-flut hormone controls delivered locally to the MMTV-*Neu* mammary glands did not modify mammary tumor development. (*A*) For tumor latency, E_2_, methyl T, OH-flut, and control groups were not significantly different (one-way ANOVA; control, *n *= 53; E_2_, *n *= 48; methyl T, *n *= 54; OH-flut, *n *= 53; mean ± SE). (*B*) Maximal incidence of mammary cancer was significant (*p* < 0.05, chi-square test). Based on group pairs analyzed separately by the Fisher’s exact test, this significance may be due to differences between the methyl T and OH-flut groups (*p *< 0.03), but no treatment was significantly different from the control group (control, *n *= 57; E_2_, *n *= 56; methyl T, *n *= 57; OH-flut, *n *= 66). (*C–E*) Survival curves for the percentage of tumor-free animals at the designated ages are depicted for control mice compared to (*C*) OH-flut (5 μg/pellet; 4 pellets/mouse), (*D*) methyl T (50 ng/pellet; 4 pellets/mouse), and (*E*) E_2_ (2.5 ng/pellet; 4 pellets/mouse) groups up to maximum age (14 months), with no significant differences detected (Gehan–Breslow–Wilcoxon test).

*Mammary tumor development with localized hormone treatments.* To assess whether the hormonal activities reported for the DDE isomers are one potential reason for the tumor outcomes, three hormone controls were examined: E_2_, methyltestosterone (methyl T), and OH-flut—an estrogen, a non-aromatizable androgen, and an antiandrogen, respectively. The tested dose of E_2_ in Elvax pellets was previously reported to remain localized because it affected responses in the treated mammary gland but not the contralateral gland or uterus (Haslam 1988). OH-flut was included at a dose equal to the dose of *p,p*´-DDE as they have been reported to be equipotent (Kelce et al. 1995). Methyl T was estimated to be approximately 20-fold less potent than E_2_. These treatments were expected to affect the gland only during pellet release because these hormones do not bioaccumulate or persist after pellet depletion as DDE does, which may be the reason that E_2_, methyl T, and OH-flut did not affect tumor latency (Figure 2A). A reduced incidence of tumors in the 5-μg/pellet OH-flut group approached significance (*p* < 0.065, Fisher’s exact test; Figure 2B), but the survival curve for the antiandrogen was not significantly different from that for control mice (Figure 2C). No effect was observed with 50-ng/pellet methyl T because its survival curve was similar to the control survival curve (Figure 2D). E_2_ (2.5 ng/pellet) shifted the curve slightly to the right, suggesting a slight delay in tumor onset although the shift was not statistically significant (Figure 2E). However, after 300 days, the E_2_ curve mimics the OH-flut (Figure 2C) and *o,p*´-DDE curves (Figure 1D). Nonsignificant differences in tumor multiplicity were detected for the hormone and DDE groups [see Supplemental Material, Figure S1A (http://dx.doi.org/10.1289/ehp.1104327)]. Additionally, mammary tumors in the seven groups had similar pathologic findings (see Supplemental Material, Figure S2).

Because the MMTV promoter is regulated by androgens (Cato et al. 1987), *Neu* expression was examined in adult mammary tissue after 2 months of treatment [see Supplemental Material, Figure S1B–C (http://dx.doi.org/10.1289/ehp.1104327)] to determine if the earlier latency could result from aberrant transgene stimulation by DDE. Transgene expression with *p,p*´-DDE and/or *o,p*´-DDE treatment was not significantly different from that of control mice. Additionally, expression of the endogenous mouse *Neu* gene was not significantly different between the control and treatment groups, suggesting DDE would not alter HER2/*Neu* expression to affect breast cancer risk in women.

*Treatment effects on metastatic progression.* No significant difference in the incidence of metastatic lung lesions—assessed histopathologically or by the number of lesions/mouse—was observed for any treatment group compared with control animals [see Supplemental Material, Figure S1D–F (http://dx.doi.org/10.1289/ehp.1104327)]. The time for tumor growth did not vary between the groups; thus, each group had similar time for the metastatic process to occur. The lack of effect on metastatic progression may be related to the pellet depletion during tumor growth; consequently, it is unknown if DDE would affect tumor aggressiveness with sustained exposure.

## Discussion

*Early onset of mammary cancer.* Developmenal exposure to *p,p*´-DDE and *o,p*´-DDE accelerated mammary tumor onset in MMTV-*Neu* mice; but, the effect was stronger and sustained until maximum age only with *p,p*´-DDE. The unaffected expression of the *Neu* transgene verifies that the tumor outcomes are related to DDE treatments and not to model-specific MMTV regulation. Consequently, *p,p*´-DDE, the most abundant and ubiquitous DDT congener, may similarly affect breast cancer development in other animal models and in women. With the short lifespan of mice, the weeks earlier onset of cancer could equate to many years in women.

In MMTV-*Neu* mice, maximal tumor incidence was not modified, suggesting that DDE acts as a tumor promoter versus initiating cancer. The lack of increased incidence correlates with most epidemiological studies examining DDT and breast cancer risk. However, in young women, DDT levels were associated with breast cancer risk before 50 years of age (Cohn et al. 2007). Because breast cancer before age 50 precedes the average age of diagnosis, the increased risk with higher levels of *p,p*´-DDT suggests an effect on latency similar to that observed with *p,p*´-DDE and *o,p*´-DDE in MMTV-*Neu* mice.

The age of onset profoundly affects breast cancer; early-onset (premenopausal) cancer (beginning at ≤ 50 years of age) is more aggressive and has a worse prognosis than breast cancer detected in postmenopausal years (Benz 2008). Inducing an earlier onset of breast cancer can increase the likelihood of women developing ER-negative tumors (Benz 2008; Vetto et al. 2009), including HER2-positive breast cancer, which this mouse model mimics. Additionally, a case–control study correlated *p,p*´-DDE concentrations with breast cancer aggressiveness (Demers et al. 2000). Therefore, a DDE-induced accelerated onset could substantially reduce the number of cancer-free years for women and adversely affect their prognosis after diagnosis.

Higher levels of *p,p*´-DDE have been detected in African Americans than in Caucasians (Snedeker 2001; Wolff et al. 2005). Although higher *p,p*´-DDE levels do not always correlate with increased breast cancer risk (Gatto et al. 2007; Millikan et al. 2000; Wolff et al. 2000a), these epidemiological studies did not investigate age of onset. Because breast cancer occurs at younger ages and is more aggressive in African-American women (Newman 2005), their early-onset breast cancer correlates well with the accelerated onset in mice exposed to *p,p*´-DDE and *o,p*´-DDE, suggesting that some populations may be more susceptible to DDE exposure.

Because the first tumor in the *p,p*´-DDE group was detected at 3 months of age and would have required many weeks to develop, treatment effects must have occurred in the immature mammary gland. This timing parallels early-onset breast cancer because diagnosis in younger women reflects early-life events affecting the immature mammary epithelium (Benz 2008). The DDE-induced tumor outcomes are also in accord with the higher risk of early-onset breast cancer detected in women with DDT exposure before 14 years of age (Cohn et al. 2007). The MMTV-*Neu* mice were exposed to DDE during a critical window during which the gland is more susceptible to hormonal and environmental influences (Cohn 2011; Fenton 2006) because the pellets were implanted before puberty and levels then accumulated during gland development. The timing of DDE exposure appears crucial for shortening tumor latency, especially for the more labile *o,p*´-DDE and, therefore, may need to be considered when associating DDT with breast cancer risk.

*DDE concentrations and breast cancer risk.* Localized delivery of *p,p*´-DDE resulted in *p,p*´-DDE concentrations in young and aged mice that are within the ranges detected in the human breast (Falck et al. 1992; Guttes et al. 1998; Ociepa-Zawal et al. 2010) and serum/plasma (Cohn et al. 2007; Eskenazi et al. 2009; Snedeker 2001), as we predicted when selecting the 5-μg/pellet dose. Therefore, accelerated tumor development occurred with doses relevant to exposure in women. The low or undetectable levels of *p,p*´-DDE in aged mice highlights its rapid metabolism, which appears to have a half-life comparable to the 80- to 120-day half-life in rats (Muhlebach et al. 1991), which is short compared with 13 years in humans (Snedeker 2001). Furthermore, *p,p*´-DDE levels in aged mice were unrelated to tumor outcomes, unlike levels in young mice before tumor detection. Considering that levels in young mice were measured within a week of detecting the first *p,p*´-DDE tumor, that tumor development requires weeks, and that DDE bioaccumulates while the pellets are releasing, the measured levels are likely higher than the levels that induced the early tumor onset. Therefore, concentrations lower than those in Table 1 may be able to accelerate mammary cancer in the mice and, possibly, also in women.

For *o,p*´-DDE, the levels detected in the *o,p*´-DDE and 2:1 ratio groups would not be detected in women because of its less persistent nature. Although rarely examined, when measured, *o,p*´-DDE is only detected in serum in a small percentage of participants and is a minor component of the DDT mixture, unlike the ubiquitous *p,p*´-DDE (Charlier et al. 2004; Dorgan et al. 1999). The lower persistence of *o,p*´-DDE is evident when comparing the lower serum:adipose concentrations in young mice and mammary fat pad levels in young versus aged mice relative to the more stable *p,p*´-DDE isomer (Tables 1–2). The rapid loss of *o,p*´-DDE is likely responsible for the earlier latency effects disappearing after 10 months of age; although other factors, such as reproductive aging with longer estrous cycling, may also contribute. Nonetheless, the ability of *o,p*´-DDE to shorten tumor latency suggests that estrogenic DDT congeners may affect breast cancer risk.

Lipid-adjusted serum levels are considered representative of exposure in breast fat; arithmetic means ranged between 5.6:1 and 3.5:1 for breast adipose:serum ratios for *p,p*´-DDE levels in women (Lopez-Carrillo et al. 1999). In young mice, with only one pool, means are not available. However, the range for arithmetic means approximates the 8:1 ratio for mammary adipose:serum in mice, suggesting that *p,p*´-DDE mouse serum concentrations can be correlated to breast cancer epidemiological studies. In multiple studies finding no correlation between lipid-adjusted *p,p*´-DDE serum/plasma levels and breast cancer risk, the serum concentration in young mice associated with early tumor onset (i.e., 230 ng/g) was within the control group concentration range (first tertile/quartile/quintile) used for determining if higher *p,p*´-DDE levels increased risk in older women (Dorgan et al. 1999; Gammon et al. 2002; Gatto et al. 2007; Laden et al. 2001; Millikan et al. 2000; Wolff et al. 2000a, 2000b, 2005). Therefore, lower concentrations than previously used may need to be considered as the reference value for *p,p*´-DDE exposure in women.

The *p,p*´-DDE serum concentration in young mice was also within the first tertile in a study reporting an increased risk of lymph-node invasive breast tumors with *p,p*´-DDE levels above 250 ng/g (Demers et al. 2000). Thus, the concentration in mice is near or below those previously determined to modify breast cancer aggressiveness. Additionally, the mouse serum concentration was at or below the mean for pregnant and reproductive age women in the 1990s and 2000s for most reported locations (Eskenazi et al. 2009), showing that mouse *p,p*´-DDE levels are within the range of current exposure in young adult women.

The data from our study suggest that DDE stored adjacent to mammary epithelium affects cancer development. However, detection of circulating DDE suggests tissues outside the mammary gland may also contribute to the early cancer onset through disrupted hormonal or non-hormonal responses. Local and systemic *p,p*´-DDE exposure in the mice correlates with human exposure, so both types of exposure may also affect breast cancer risk in women.

*Hormonal actions of DDE.* Accelerated tumor development by DDE suggests it affects tumor promotion, a stage of carcinogenesis affected by hormones, and not initiation, as would occur with genotoxic or DNA damage mechanisms that have been speculated with increased breast cancer risk from early DDT exposure (Cohn et al. 2007). Accordingly, the endocrine-disrupting properties of *p,p*´-DDE and *o,p*´-DDE may have affected the tumor outcomes. However, the E_2_, methyl T, and OH-flut hormone controls did not modify tumor onset. Even without correlation to these treatments, the antiandrogenic and estrogenic actions of the DDE isomers could influence latency via dose effects, combined effects with non-hormonal actions, and/or unique, hormone-dependent responses that are not induced by the tested hormone controls.

As estrogen exposure in immature glands can subsequently suppress mammary carcinogenesis in 7,12-dimethylbenz[*a*]anthracene (DMBA)-treated rats (Cabanes et al. 2004), the delayed tumor onset with *o,p*´-DDE and E_2_ in mice > 10 months of age suggests that *o,p*´-DDE may have estrogenic actions during mammary gland development. However, *p,p*´-DDE does not result in a similar delayed onset even though it is reported to weakly bind and transactivate the estrogen receptor (Andersen et al. 1999; Kelce et al. 1995; Payne et al. 2001). Proliferation assays in MCF-7 cells suggest *p,p*´-DDE has weak estrogenic activity (Andersen et al. 1999; Payne et al. 2001); however, antiandrogenic actions, which induce enhanced estrogen activation, may be misconstrued as estrogenic. For example, in MMTV-*Neu* mice, the OH-flut survival curve (Figure 2C) after 10 months of age was similar to the E_2_ (Figure 2E) and *o,p*´-DDE (Figure 1D) curves. OH-flut may reduce androgen effects in the immature mammary gland as androgens are required for normal gland development (Yeh et al. 2003) and suppress estrogenic action in mammary tissue (Liao and Dickson 2002). Accordingly, *p,p*´-DDE may not have estrogenic or antiandrogenic activity as its survival curve differs from E_2_, *o,p*´-DDE, and OH-flut. Further studies are required to determine if the hormonal properties of *p,p*´-DDE affect the mammary gland and are related to the tumor outcomes.

*Mixtures of* p,p*´-DDE plus* o,p*´-DDE.* The inability of the 2:1 ratio to affect latency was unexpected because both individual DDE isomers accelerated tumor onset. If both isomers had similar activity (e.g., estrogenic or antiandrogenic activity), an additive effect would be expected for the combination as their concentrations (3.3 + 1.7 μg/pellet) equal the 5-μg/pellet dose of the individual congeners. Because no latency effect occurred with the 2:1 ratio, the DDE isomers must not complement each other’s actions and thus may not have similar hormonal or non-hormonal activities. Therefore, the loss of effect with the combination may be due to the lower doses of the individual DDE compounds or to one isomer counteracting the actions of the other to block its cancer-promoting properties. However, because of the infrequent and low *o,p*´-DDE exposure in humans, the relevance of these findings with this mixture is uncertain.

In the study that correlated high serum levels of *p,p*´-DDT but not *p,p*´-DDE in young women with an increased breast cancer risk before 50 years of age, the mean *p,p*´-DDE levels were > 20-fold higher than in young mice (Cohn et al. 2007). Not correlating high *p,p*´-DDE exposure with increased risk does not preclude it from influencing breast cancer development; rather it suggests that, at the concentrations examined, *p,p*´-DDE was not a good marker for evaluating the risk or timing of DDT exposure. Although the reference tertile of Cohn et al. (2007) for *p,p*´-DDE may be too high, it is also possible that the levels and/or ratios of both *p,p*´-DDT and *p,p*´-DDE are important. That is, higher levels of *p,p*´-DDT in combination with *p,p*´-DDE could enhance the risk of early-onset breast cancer in comparison to women with lower *p,p*´-DDT levels. Although our results highlight different DDT metabolites in mammary carcinogenesis, overall, our findings support and reinforce the pivotal study in young women demonstrating that early exposures to DDT can affect breast cancer risk (Cohn et al. 2007).

## Conclusion

Our study directly links DDE exposure to early onset mammary cancer. These data suggest that locally stored DDE promotes, but does not cause, mammary tumorigenesis. Because the MMTV-*Neu* model mimics HER2-positive cancer, DDE exposure may only affect specific types of breast cancer such as HER2-positive or ER-negative breast cancer, which are more common in younger patients with early onset breast cancer. Our findings indicate future epidemiological studies may need to consider age of exposure and of tumor onset for examining correlations between *p,p*´-DDE and breast cancer risk. Millions of women are coming of age for developing breast cancer associated with exposure decades earlier and, therefore, are still at risk. The results in the mice suggest that even without further exposure to *p,p*´-DDE, the risk of accelerated breast cancer onset could already be imprinted into adult women’s breast tissue. Consequently, the declining levels of environmental and *in vivo p,p*´-DDE may be important for future generations of young girls, but not for women already exposed in their youth before levels declined. Whether the levels have declined sufficiently in countries where DDT use is banned is unknown as the safe limits of exposure have not been determined. However, because of the prevalence and persistence of *p,p*´-DDE in humans, animals, and the environment, the findings in this study on a life-threatening disease renew concerns regarding the continued use of DDT and exposures from its previous use.

## Supplemental Material

(397 KB) PDF
