# Supplementary material for: Accelerated Mammary Tumor Onset in a HER2/Neu Mouse Model Exposed to DDT Metabolites Locally Delivered to the Mammary Gland
Source: Environ Health Perspect. 2012 Apr 18;120(8):1170–6. doi: 10.1289/ehp.1104327 (PMC3440075; doi:10.1289/ehp.1104327)
Supplement: (397 KB) PDF [file ehp.1104327.s001.pdf]

Accelerated Mammary Tumor Onset in a HER2/*Neu* Mouse Model Exposed to DDT Metabolites Locally Delivered to the Mammary Gland

Nakpangi A. Johnson, Arline Ho, J. Mark Cline, Claude L. Hughes,
Warren G. Foster, and Vicki L. Davis

Table of Contents

Supplemental Material, Methods page 2

Supplemental Material, Figure S1 page 4

Supplemental Material, Figure S2 page 6

Supplemental Material, References page 8

Supplemental Material, Methods

Histopathology

Mammary tumors, adjacent mammary tissue (if present), and lungs were fixed with cold 4% paraformaldehyde, embedded in paraffin, cut into 6 μm sections, and stained with hematoxylin and eosin. Sectioning and staining were performed by Mass Histology Service (Worcester, MA). Histopathology for assessing metastatic cancer incidence in the mouse lungs by our board-certified veterinary pathologist (J.M.C.) was performed as previously described (Davis et al. 2008).

Animal Study for Pre-tumor Endpoints

Additional MMTV-*Neu* mice (n=20/group) were bred and treated for analysis of early endpoints in adult females after 2 months of treatment. Real-time RT-PCR for *Neu* expression was performed as previously described with primers specific to the mouse and rat (transgene) *Neu* genes (Davis et al. 2008). To verify the animals were prepubertal at the time of implant surgery, vaginal opening was examined in these additional animals; it was detected between ages 20 and 26 days or between 1-4 days after surgery. All surgeries for the tumor study animals occurred by age 26 days, with 95% of the mice undergoing pellet implantation by 24 days of age. Only a rudimentary mammary ductal structure would be present in prepubertal female mice as evidenced in the activated MMTV-*Neu* model (Hewitt et al. 2002). Vaginal smears stained with Dif-stain kit (IMEB Inc., San Marcos, CA) were performed to confirm the mice treated for 2 months were in estrus at necropsy.

DDE Concentrations in Serum and Mammary Fatpads

Frozen pooled samples of serum and mammary glands were shipped with blinded labeling to Centre de Toxocologie at the Institut National de Santé Publique Quebec (INSPQ; Quebec's Institute of Public Health) for analysis. Samples were analyzed for DDE residues according to INSPQ Method E-446. Samples were enriched with the internal standard, *p,p'*-DDE (ring-$^{13}C_{12}$, 99%; CIL-CLM-1627, Cambridge Isotope Laboratories, Inc.), and denatured with formic acid. Polar extracts were cleaned on Florisil columns prior to gas chromatography-mass spectroscopy (GC-MS; Agilent 6890) as recommended by U.S. EPA methods for pesticide residues (U.S. Environmental Protection Agency 1980). The Florisil is purified with dichloromethane on a soxhlet extraction system running at 60 °C for 6 hours (60 washing cycles) and then deactivated with 0.5% water. Ions generated were measured after negative chemical ionization with an electron capture detector (Agilent G2397A) and mass detector (Agilent 5973 Network) coupled to the GC-MS and analyzed with Agilent MSD Chem software. Concentrations were evaluated using the percent recovery of the labeled internal standard. The detection limit for *p,p'*-DDE is 0.09 μg/L with 14% reproducibility (0.01 μg/L for *o,p'*-DDE).

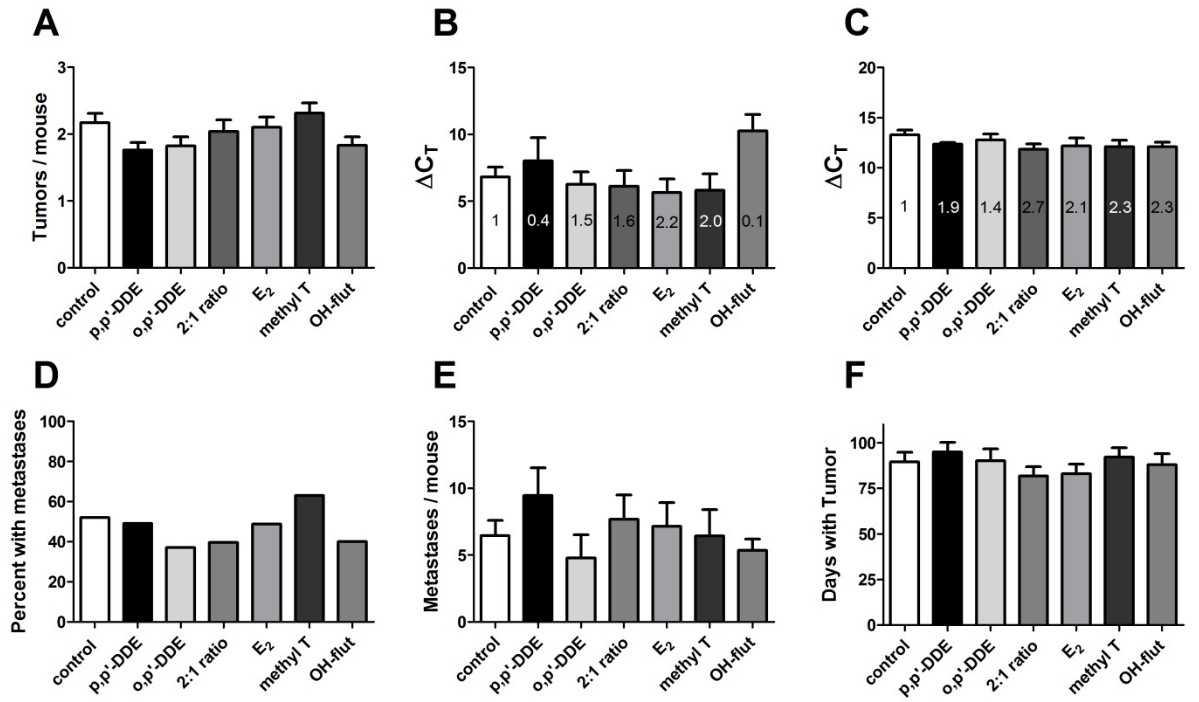

Supplemental Material, Figure S1. Tumor multiplicity, transgene and endogenous *Neu* expression, and mammary tumor metastatic incidence were not modified by DDE exposure. (**A**) Mammary tumor multiplicity designated by the mean number of tumors/mouse was significant (p=0.043, one-way ANOVA), but no significant differences between the groups were detected (Tukey's test). (**B**) The expression of the rat *Neu* transgene in mammary tissue from the MMTV-*Neu* female mice treated for 2 months was determined by real-time RT-PCR. The ΔC_T value represents the normalized threshold cycle (C_T) for the *Neu* gene relative to the cyclophilin A (*ppia*) housekeeping gene. The fold difference compared to the control group determined by the $2^{-\Delta\Delta Ct}$ method is shown within the bar for each group (note that lower ΔC_T values represent higher expression). No significant differences were found (one-way ANOVA; n=4-9). However, the antiandrogenic action of OH-flut was suggested by reduced *Neu* expression, which approached significance compared to control mice (p< 0.06, Mann-Whitney test). It is possible

this inhibition is due to antiandrogen and/or antiprogestin activity, but intact females would have higher endogenous progesterone, which also regulates MMTV promoter (Cato et al. 1987), than androgen levels to stimulate *Neu* expression. *p,p'*-DDE has antiprogestin action (Li et al. 2008; Klotz et al. 1997) and non-significantly inhibited rat *Neu* expression. However, as *Neu* mRNA levels were measured 2 months after treatment, any effects of OH-flut and *p,p'*-DDE may also be due to indirect as well as direct effects on *Neu* expression. (**C**) The endogenous mouse *Neu* gene expression by real-time RT-PCR was not significant (one-way ANOVA; n=6-9), with the fold difference versus the control group ($2^{-\Delta\Delta Ct}$ method) shown within each bar. (**D**) For all female mice with mammary tumors and with lungs examined by histopathology, the incidence of micrometastases was not significant by chi-square test (control, n=50; *p,p'*-DDE, n=53; *o,p'*-DDE, n=46; 2:1 ratio, n=48; E_2, n=43; methyl T, n=46; OH-flut, n=50). (**E**) The mean number of micrometastases within the blood vessels and/or invading into the lung tissue detected in the lung sections per mouse for each group was not significantly different (one-way ANOVA test). (**F**) The number of days from tumor detection to death for animals with metastatic lesions was not significantly modified by the treatments (one-way ANOVA test). Mean ± SEM is shown for panels A-C, and E-F; $p<0.05$ considered significant. E_2, 17β-estradiol; methyl T, methyltestosterone; OH-flut, hydroxyflutamide; 2:1 ratio, *p,p'*-DDE:*o,p'*-DDE.

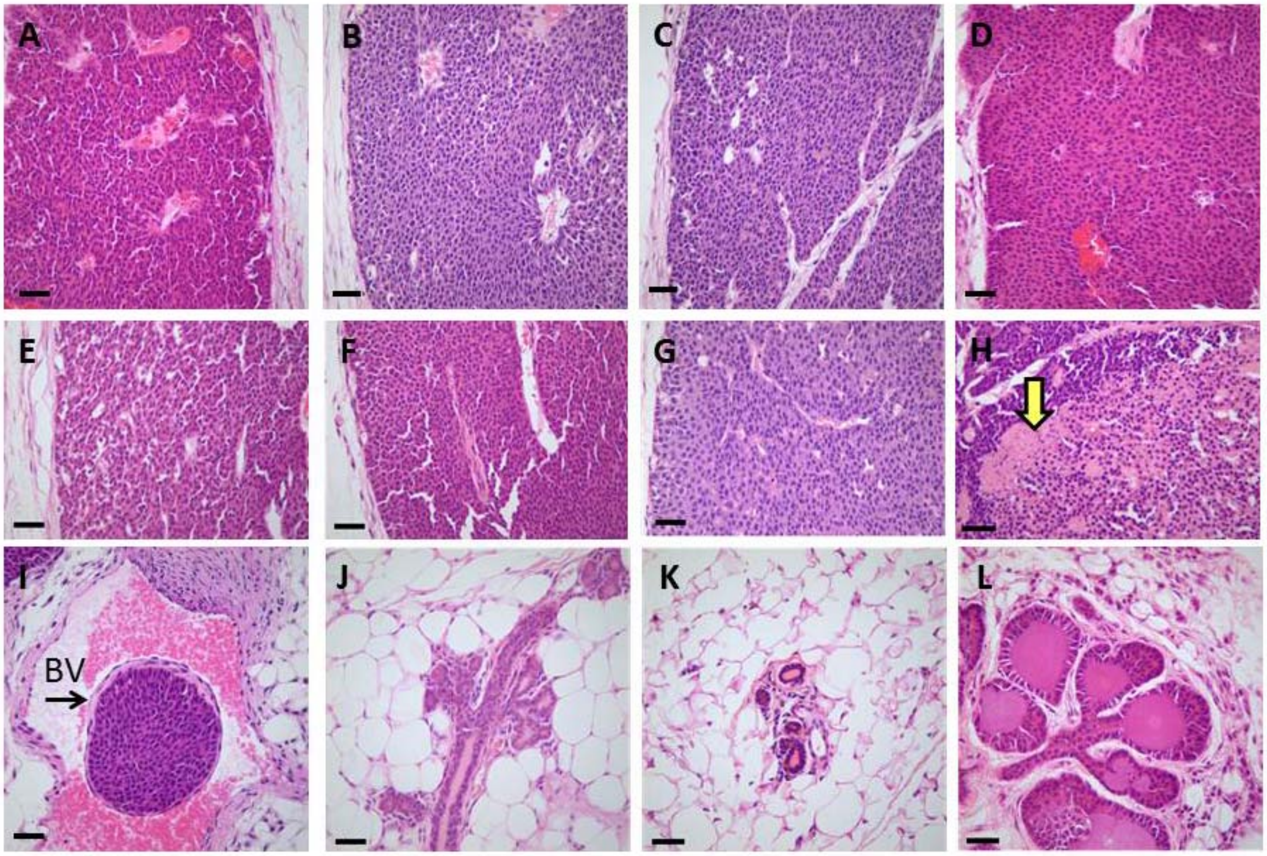

Supplemental Material, Figure S2. MMTV-*Neu* mice develop mammary adenocarcinomas in control and treated groups while adjacent mammary tissue is normal or has pre-malignant changes. Representative normal, precancerous, and malignant mammary tissues are shown from hematoxylin and eosin-stained sections from control and treated mice. Mammary masses in all 7 groups were identified as adenocarcinomas by histopathology (100% of tumors analyzed). Panels A-G are mammary tumors from the following groups: (**A**) Control; (**B**) *p,p'*-DDE; (**C**) *o,p'*-DDE; (**D**) 2:1 ratio; (**E**) E_2; (**F**) methyl T; and (**G**) OH-flut. (**H**) Necrosis is present in some tumors from all groups, such as shown in this *o,p'*-DDE mammary tumor (arrow points to large area of necrosis). (**I**) Vascular invasion (intravasion) is detected within some tumors in every group; tumor cells fill the blood vessel (BV) within the mammary adenocarcinoma (*p,p'*-DDE group). Panels J-L: For mammary tumors that did not encompass all of the mammary gland,

adjacent mammary tissue was also examined. No abnormalities were noted except in 2 of 35 tumors with adjacent mammary tissue on the section (n=3-7/group): one animal in the 2:1 ratio group had precancerous lesions and ductal hyperplasia and one mouse in the OH-flut group had precancerous changes. Normal mammary tissue adjacent to (**J**) an OH-flut and (**K**) methyl T tumor are displayed. (**L**) Precancerous changes evident in the mammary tissue adjacent to a 2:1 ratio tumor. The bar in each photograph equals 50 microns.

Supplemental Material, References

Cato AC, Henderson D, Ponta H. 1987. The hormone response element of the mouse mammary tumour virus DNA mediates the progestin and androgen induction of transcription in the proviral long terminal repeat region. EMBO J 6:363-368.

Davis VL, Jayo MJ, Ho A, Kotlarczyk MP, Hardy ML, Foster WG, et al. 2008. Black cohosh increases metastatic mammary cancer in transgenic mice expressing c-erbB2. Cancer Res 68:8377-8383.

Hewitt SC, Bocchinfuso WP, Zhai J, Harrell C, Koonce L, Clark J, et al. 2002. Lack of ductal development in the absence of functional estrogen receptor alpha delays mammary tumor formation induced by transgenic expression of ErbB2/neu. Cancer Res 62:2798-2805.

Klotz DM, Ladlie BL, Vonier PM, McLachlan JA, Arnold SF. 1997. *o,p'*-DDT and its metabolites inhibit protesterone-dependent responses in yeast and human cells. Mol Cell Endocrinol 129:63-71.

Li J, Li N, Ma M, Giesy JP, Wang Z. 2008. In vitro profiling of the endocrine disrupting potency of organochlorine pesticides. Toxicol Lett 183:65-71.

U. S. Environmental Protection Agency. 1980. Manual of Analytical Methods for the Analysis of Pesticides in Humans and Environmental Samples. U. S. Environmental Protection Agency, Washington, D.C.
